# Sustainable Recovery of Phenolic Compounds from Distilled Rosemary By-Product Using Green Extraction Methods: Optimization, Comparison, and Antioxidant Activity

**DOI:** 10.3390/molecules28186669

**Published:** 2023-09-17

**Authors:** Maria Irakli, Adriana Skendi, Elisavet Bouloumpasi, Stamatia Christaki, Costas G. Biliaderis, Paschalina Chatzopoulou

**Affiliations:** 1 Hellenic Agricultural Organization—Dimitra, Institute of Plant Breeding and Genetic Resources, 57001 Thessaloniki, Greece; antrsken@teiemt.gr (A.S.); elisboul@abo.ihu.gr (E.B.); stamchri@agro.auth.gr (S.C.); 2 Department of Food Science and Technology, School of Agriculture, Aristotle University of Thessaloniki, 54124 Thessaloniki, Greece; biliader@agro.auth.gr

**Keywords:** rosemary solid waste material, microwave-assisted extraction, ultrasound-assisted extraction, accelerated solvent extraction, Soxhlet, phenolics, antioxidant capacity, LC-MS, response surface methodology

## Abstract

Rosemary solid distillation waste (SWR), a by-product of the essential oil industry, represents an important source of phenolic antioxidants. Green technologies such as ultrasound-assisted extraction (UAE), microwave-assisted extraction (MAE), and accelerated solvent extraction (ASE) of phenolic compounds from SWR were optimized as valorization routes to maximize yield, rosmarinic acid (RMA), carnosol (CARO) and carnosic acid (CARA) contents. Response surface methodology was used in this context, with ethanol concentration (X_1_), extraction temperature (X_2_), and time (X_3_) being the independent variables. A second-order polynomial model was fitted to the data, and multiple regression analysis and analysis of variance were used to determine model fitness and optimal conditions. Ethanol concentration was the most influential extraction parameter, affecting phenolic compounds, while the influence of other parameters was moderate. The optimized conditions were as follows: X_1_: 67.4, 80.0, and 59.0%, X_2_: 70, 51, and 125 °C, and X_3_: 15, 10, and 7 min for MAE, UAE, and ASE, respectively. A comparison of optimized MAE, UAE, and ASE with conventional Soxhlet extraction techniques indicated that ASE provided a higher extraction yield and content of phenolic compounds. However, UAE represented the best process from an environmental point of view, allowing an improved extraction of phenolics from SWR with high energy efficiency and low energy costs.

## 1. Introduction

*Rosmarinus officinalis* L. (rosemary) is an aromatic plant native to the Mediterranean region, cultivated worldwide, and used as a condiment in various products or as an essential oil (EO) or extract. In addition, rosemary is considered a medicinal plant due to its health benefits [1]. Rosemary and its derived products find extensive application in foods and beverages, pharmaceuticals, cosmetic products, as well as home-care and cleaning products. Rosemary’s potential applications are mainly due to its EO and the high content of phenolic compounds, which exhibit bioactive properties such as antioxidant, antifungal, antibacterial, anti-inflammatory, etc., with beneficial effects on human health. Antioxidant phenolic compounds from rosemary are reported to improve the antioxidant defense and attenuate oxidative stress in rats by boosting the activity of antioxidant enzymes, reducing the amount of thiobarbituric acid reactive substances and improving the serum lipid profile, contributing to the prevention of cardiometabolic risk [2].

In addition, phenolics with antioxidant activity from rosemary have been reported as food additives and flavoring agents [3] and as natural preservatives able to prevent microbial growth and food spoilage [4]. Further to their beneficial effects on human health, the demand of the food industry for natural antioxidants and preservatives makes phenolic extracts from rosemary an attractive, functional ingredient for food formulations. Rosemary plant extract was approved as a natural antioxidant and assigned the E-392 number (European Union Directives 2010/67/EU, 2010/69/EU, and 2012/231/EU) [5,6,7].

Although rosemary is rich in many phenolics with high antioxidant capacity, high variability of these compounds may occur in the raw plant materials of different origins due to several factors, such as genetics and environment-agronomic practices, as well as the extraction method employed for their recovery. Rosemary phenolic compounds belong to different chemical classes (phenolic acids, flavonoids, diterpenes, etc.), with rosmarinic acid (RMA) representing the most abundant phenolic acid in methanolic extracts of rosemary leaves [8]. Additionally, rosemary leaves contain high amounts of phenolic diterpenes, such as carnosic acid (CARA) [9,10], and lesser amounts of carnosol (CARO), which is considered the main oxidation product of CARA [9]. RMA and CARA are known bioactive compounds with antimicrobial activity [11]. Among the phenolics, CARA is known for its antioxidant activity, which is even higher than that of the most commonly used synthetic antioxidants, BHT and BHA, but its instability in the presence of oxygen results in different breakdown compounds depending on the source (matrix) and the extraction method [12].

The extensive use of rosemary has rapidly increased its worldwide demand, especially for its essential oil, generating high amounts of post-distillation solid waste material. The accumulation and limited use of these solid by-products, mainly as fertilizer and less as an ingredient in animal feed, results in environmental problems [13]. As these residues are rich in bioactive substances, they can be exploited in numerous ways, supporting sustainable agricultural production practices. In an early study, Almela et al. [14] analyzed the antioxidant activity of a distilled rosemary by-product extract and compared it with that of crude fresh rosemary extracts. The results revealed that crude-fresh rosemary exhibited antioxidant activity almost identical to pure δ-tocopherol and higher than BHT, while extracts prepared from distilled rosemary by-products showed the lowest activity. Recently, the solid waste of rosemary (SWR) derived from essential oil production with steam distillation was confirmed as a rich source of phenolic compounds [15,16].

In addition to natural variation, the amount of phenolics in the rosemary extracts varies depending on the type of process used to obtain essential oil [16] as well as on the raw material pretreatment and extraction process applied [17]. Among the extraction parameters, solvent polarity is an important factor that highly affects the extractability of different phenolics since it can favor or impede the extraction of single phenolic compounds from a specific plant material. Moreover, previous studies indicate that aqueous mixtures of ethanol and acetone exhibit improved solvency than their respective pure solvents, resulting in rich phenolic content extracts from SWR [18]. The amount of RMA, CARO, and CARA was much higher in the study of Irakli et al. [15], which used 70% methanol, compared to the study of Psarrou et al. [18] that used 60% ethanol, and to the study of Almela et al. [14], where pure methanol was used for extraction of phenolic compounds from SWR. In food applications, only those solvents generally recognized as safe (GRAS) must be used, and the authorized specific limits in concentration should be taken into consideration.

Although maceration is a conventional and widely used solid–liquid extraction technique for obtaining antioxidants from rosemary tissues [19] because of its simplicity, easiness to scale up, and low cost, it is characterized by prolonged extraction times, substantial amounts of solvents, many extraction steps, and higher energy consumption. Soxhlet extraction is widely used at the lab scale for recovering phenolic-rich extracts. This method uses solvents with high hydrogen-bonding abilities (such as methanol and acetone) [20,21], resulting in high yields of antioxidant compounds, especially phenolic diterpenes. Nevertheless, many drawbacks exist due to the high temperatures and prolonged processing time, low selectivity, and issues linked with eliminating solvent residues as directed by food regulations. Thus, many phenolics decompose, resulting in extracts with poor phenolic profile and limited bioavailability.

Although conventional extraction methods result in extracts rich in phenolics and exerting substantial antioxidant activity, more selective extraction techniques are preferred nowadays that are more environmentally friendly. New “green” extraction techniques that provide higher efficiency, lower energy, and solvent consumption have already been utilized in extracting antioxidants from rosemary. The literature reports an improvement in rosemary extraction processes, efficiency, and increased final product quality by using alternative techniques like ultrasound-assisted extraction (UAE) [15,18,22], microwave-assisted extraction (MAE) [23,24], supercritical fluid extraction (SFC) [25,26], pressurized liquid extraction (PLE) [26], water extraction and particle formation on-line (WEPO) extraction [26], and accelerated solvent extraction (ASE) [27].

To date, there are only a few studies focusing on the bioactive composition of SWR extracts, mainly applying UAE [16,18,28]. At the same time, there are no reports demonstrating the influences of other “green” extraction methods like MAE and ASE on the yield, phenolic composition, and antioxidant activity of SWR extracts. Thus, further research on the development of eco-friendly and sustainable extraction approaches for phenolic recovery from SWR is necessary.

Optimizing an extraction process has advantages in terms of energy, time, and solvent consumption and is strongly recommended even if the plant material has undergone different pretreatments that may cause alteration in the matrix structure and composition of the plant tissues [29]. Thus, an appropriate choice of extraction technique and process parameters (solvent, temperature, time, and solid-to-solvent ratio) must be determined to efficiently obtain a high yield of bioactive compounds with antioxidant activity from SWR for their further use as food additives.

The present study aimed to investigate the effectiveness of three green extraction techniques (MAE, UAE, and ASE) for the recovery of bioactive phenolics from SWR. An optimization study of the extraction parameters for each technique separately utilizing Response Surface Methodology (RSM) was performed to accomplish this aim. Moreover, the efficiency of green extraction techniques was compared against a traditional extraction method (Soxhlet extraction). The efficiency of each extraction technique was estimated in terms of the concentration of the main phenolics, such as RMA, CARA, and CARO, in the obtained extracts under optimal conditions.

## 2. Results and Discussion

### 2.1. Analysis of Phenolic Compounds

SWR remained after distillation of the essential oil, which is a rich source of phenolics with strong redox properties, which are responsible for a wide range of health-related biological activities, including antibacterial, antifungal, and antioxidant functions [15,28]. This work investigated the efficacy of UAE, MAE, and ASE techniques with varying ratios of ethanol/water solvents for the rapid and selective recovery of phenolic compounds present in SWR. This solvent mixture was selected due to the greater solubility of phenolic compounds and its recognition as a safe extractant for use in the food industry [18,30]. According to previously reported data, the highest extraction yield of rosemary plant materials was noted with 80% ethanol, as the presence of water (which has a stronger dipole moment than the alcohols) destabilizes the plant cell walls, increasing the extraction of phenolic compounds [30]. Thus, the range of pure water (0% ethanol) up to 80% ethanol was chosen as the two limits of the mixed solvent in the experimentation to optimize the yield of extracted phenolics. Thus, the extraction efficiency for each technique was evaluated across a series of single-extraction steps.

**Figure 1:**
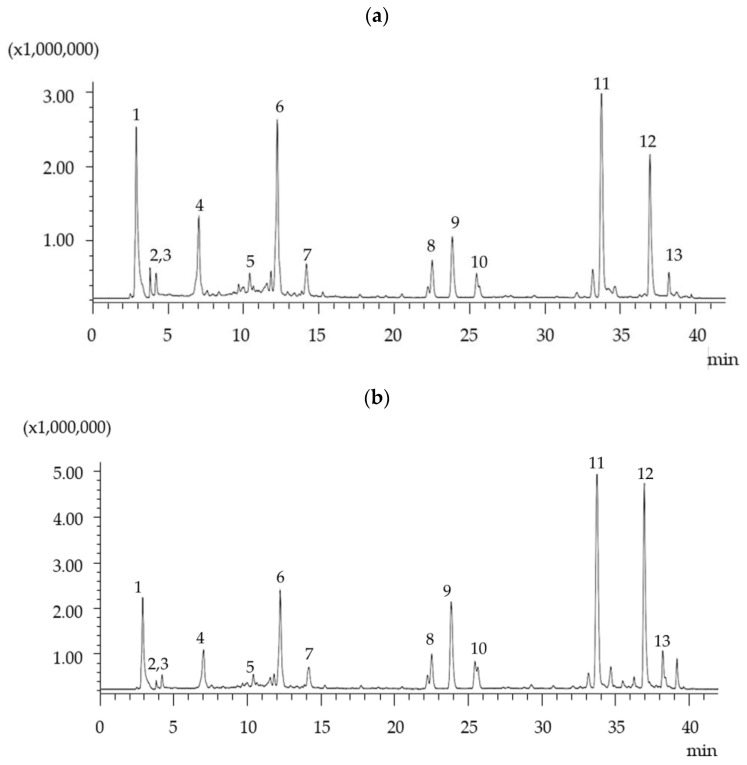
Total ion chromatogram (TIC) by negative ion mode electrospray ionization mass spectrometry (ESI/MS) of the aqueous (**a**) and 80% ethanolic (**b**) extracts of SWR obtained using ASE. Peaks on the chromatogram correspond to the following: 1, quinic acid; 2, citric acid; 3, danshensu; 4, gallocatechin isomer; 5, isorhamnetin-3-O-D-glucoside; 6, rosmarinic acid; 7, salicylic acid (internal standard); 8, 9, and 10, rosmanol isomer; 11, carnosol; 12, carnosic acid; and 13, rosmanol isomer.

HPLC analysis of ASE, MAE, and UAE extracts revealed RMA, CARA, and CARO to be the main components and several minor peaks that corresponded to flavonoid glycosides and rosmanol isomers. Identification of the various compounds of the extracts was carried out according to previous studies [15,16] and confirmed by HPLC-MS. A representative LC-MS chromatographic profile of aqueous and 80% ethanol extracts of SWR obtained using ASE at 95 °C is shown in Figure 1. As can be seen in this figure, several phenolic compounds could be separated in a total analysis time of 40 min. It is obvious that the elution of phenolic compounds in both SWR extracts by pure water and 80% ethanol extracts followed a similar pattern with differences in the yields. As can be seen in Figure 1b, at the end of the chromatogram, the 80% ethanol extracts best recovered the phenolic diterpenes, while RMA recovery seemed to be independent of ethanol concentration (Figure 1a). 

Comparing our results with the literature, Psarrou et al. [18] have reported almost the same extraction for phenolic diterpenes, while the results were different on RMA recovery, which was found to be higher on the aqueous extract than on the ethanolic extract. However, in another study, Ziani et al. [31] reported that RMA reached its maximum yield with 100% ethanol, whereas CARO and CARA were highly recovered in the aqueous extracts. Therefore, besides extraction yield (EY), the other dependent variables chosen for the optimization of MAE, UAE, and ASE techniques were the recovered amounts of RMA, CARA, and CARO.

### 2.2. Fitting, Adequacy of the Models and Optimization

Preliminary results revealed that the effect of the solid-to-solvent ratio on the phenolic content was negligible. Thus, in the Box–Behnken design, the only extraction parameters that were included were ethanol concentration (X_1_), extraction temperature (X_2_), and extraction time (X_3_). Moreover, the preliminary results revealed the range of process parameters considered for the Box–Behnken design. The software generated the mathematical models for each response variable (EY, RMA, CARO, and CARA) of each extraction technique applied (UAE, MAE, and ASE) based on the acquired experimental data. The equations presented in Table 1 contain only significant terms (*p* ≤ 0.05), except for non-significant linear terms (noted with *) for a process-independent variable when its respective quadratic or interaction terms are statistically significant.

Table 1 also reports the results of the analysis of variance (ANOVA) of the quadratic models applied to the experimental data. The F-value for the lack-of-fit was insignificant (*p* > 0.05) for each response and each extraction technique, thus being adequate for confirming the model validity in each case. On the other hand, high values of the coefficients of determination (R^2^ ≥ 88.0%), adjusted coefficients of determination (R^2^_adj_ ≥ 83.1%), and predicted coefficients of determination (R^2^_pred_ ≥ 70.4%) indicated that the equations are valid and describe the experimental data very well. Specifically, the R^2^ and R^2^_adj_ were higher than 88.0% and 83.1% for UAE, 94.6% and 93.2% for MAE, and 89.1% and 87.3% for ASE extraction techniques, respectively. The difference between the R^2^_pred_ and the R^2^_adj_ was less than 20% for each response, regardless of the extraction technique applied, suggesting that the developed models provide a good prediction of the experimental data. Moreover, each response is well predicted by the independent variables chosen. Thus, each generated equation could successfully be utilized for predicting the respective response within the tested ranges of values for the independent variables.

### 2.3. Effect of Different Process Parameters on Extraction Responses of Phenolics

Based on the resulting equations, ethanol concentration significantly influences all the responses studied (EY, RMA, CARO, and CARA). It was observed that it negatively affected only EY, while the rest of the responses were positively affected. The higher absolute value of the coefficient of the linear term of ethanol (X_1_) reveals that ethanol concentration has the highest decreasing effect on the EY in the case of the UAE process. The increase in temperature (X_2_) or extraction time (X_3_) increases the EY of UAE and MAE extracts. In the case of ASE extracts, the temperature does not affect the EY (*p* > 0.05).

**Figure 2:**
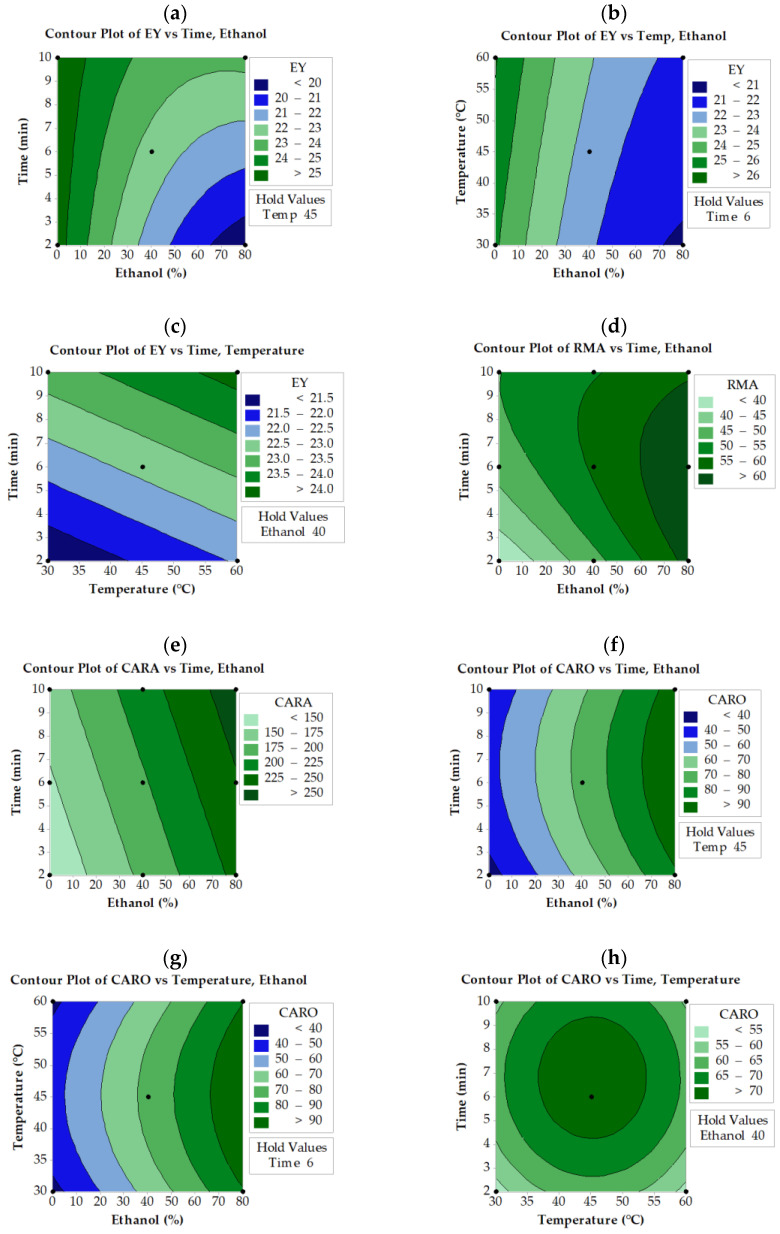
Contour plots for extraction yield (EY), rosmarinic acid (RMA), carnosol (CARO), and carnosic acid (CARA) as a function of ethanol concentration, extraction temperature, and time under ultrasound-assisted extraction (UAE). The values of the missing factor were kept at the center point, e.g., ethanol concentration 40% (Ethanol 40), time extraction 6 min (Time 6) and temperature extraction 45 °C (Temp 45).

**Figure 3:**
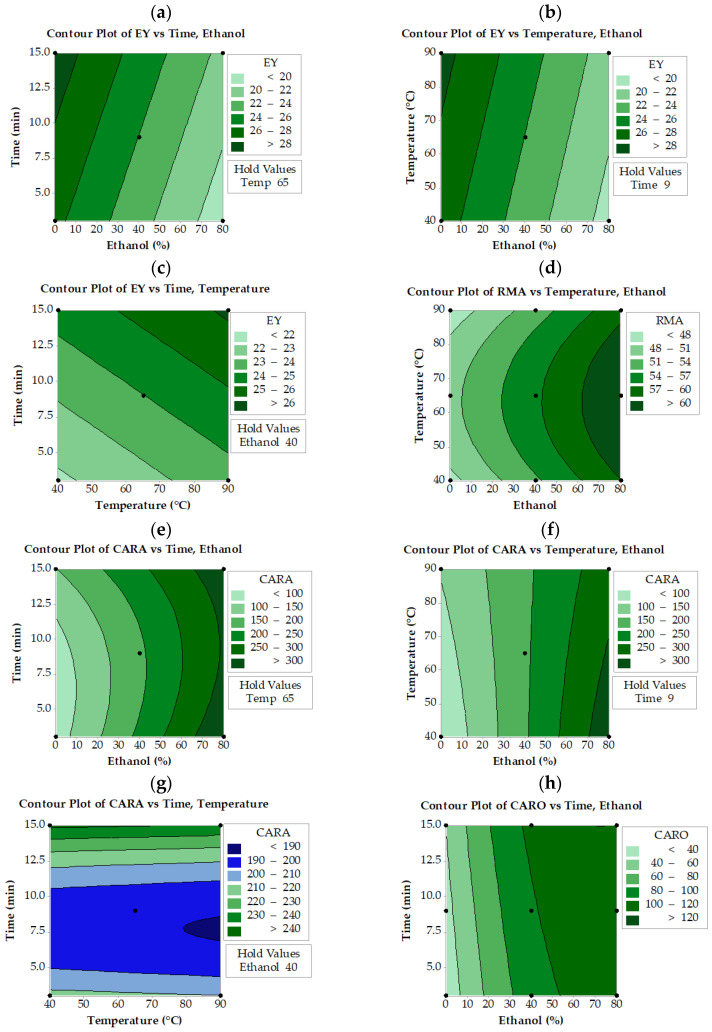
Contour plots for extraction yield (EY), rosmarinic acid (RMA), carnosol (CARO), and carnosic acid (CARA) as a function of ethanol concentration, extraction temperature, and time under microwave-assisted extraction (MAE). The values of the missing factor were kept at the center point, e.g., ethanol concentration 40% (Ethanol 40), time extraction 6 min (Time 9) and temperature extraction 45 °C (Temp 65).

**Figure 4:**
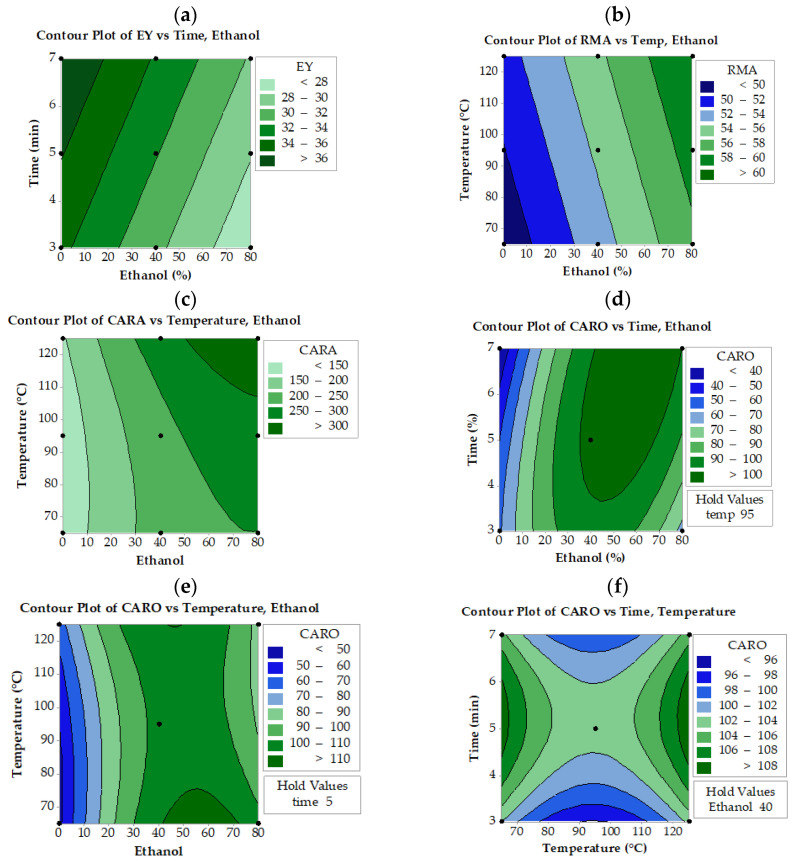
Contour plot for extraction yield (EY), rosmarinic acid (RMA), carnosol (CARO), and carnosic acid (CARA) as a function of ethanol concentration, extraction temperature, and time during accelerated solvent extraction (ASE). The values of the missing factor were kept at the center point, e.g., ethanol concentration 40% (Ethanol 40), time extraction 6 min (Time 5) and temperature extraction 45 °C (Temp 65).

It is evident from contour plots (Figure 2a–c) that higher EY values can be obtained when low ethanol concentrations with high extraction time and temperature in UAE extracts are combined. An increase in both extraction time and temperature results in a higher EY. Similar behavior was observed for MAE extracts (Figure 3a–c). Regarding ASE extracts, lower ethanol and higher extraction times result in greater EY (Figure 4a). Apparently, ASE could reach EY values of more than 36% if SWR is extracted for more than 5 min using less than 20% ethanol, levels much higher than those obtained by UAE and MAE. These results indicate that EY could increase if water concentration increases, implying that the co-extraction of compounds other than phenolics (mostly water-soluble carbohydrates, proteins, etc.) may contribute to the increased yield. The literature reports that mixtures of alcohols and water are more efficient in extracting phenolics from medicinal plants, giving a rather high yield of total extract [32]. Similarly, it has been reported that high ethanol concentrations in aqueous ethanol/water mixtures decrease the EY of the solubilized material [33]. Do et al. [34] also reported that the EY of *Limnophila aromatica* increases with decreasing ethanol concentration in aqueous media.

RMA levels of SWR extracts depend mainly on ethanol concentration and temperature of extraction, as revealed by the respective equations in Table 1 and contour plots (Figure 2d, Figure 3d, and Figure 4b). Ethanol concentration is the most significant factor that affects RMA levels in all three extraction methods, while extraction time is the second significant parameter in UAE extracts and temperature in MAE and ASE extracts. Thus, to obtain RMA values higher than 60 mg/g using MAE or UAE, the ethanol concentration should be reduced to 60% if, at the same time, the temperature is set to about 65 °C and the time to about 7 min. These settings also allow a reduction in the amount of solvent used in UAE and MAE processes. In the ASE process, similar results can be achieved if slightly higher ethanol concentrations are applied at much higher temperatures (125 °C). Generally, UAE, MAE, and ASE need more than 65% ethanol in order to achieve the highest concentration of RMA. In contrast with UAE and MAE, ASE needs higher temperatures (>80 °C) to reach the same RMA values.

As for CARA, it seems that not all factors significantly affect the extraction levels in the case of ASE and UAE processes (Figure 2e and Figure 4c). During the UAE process, CARA is affected by the ethanol concentration and time of extraction, while in ASE, it is affected by the ethanol concentration and the temperature of extraction. On the other hand, all three parameters significantly affect the CARA levels during MAE (Figure 3e–g). Higher than 300 mg/g CARA can be achieved only by simultaneously applying an ethanol concentration of more than 50% and temperatures higher than 110 °C in the case of the ASE process (Figure 3c). It appears that the UAE process was not as efficient as ASE in obtaining higher than 300 mg/g CARA within the levels tested for the three parameters. Higher than 250 mg/g extract can be achieved only by using more than 70% ethanol concentration and longer than 7 min extraction time (Figure 2e).

It seems that much higher levels (higher than 300 mg/g) are possible using MAE either at the lowest (3 min) or at the highest (15 min) extraction time in combination with the lowest temperatures but using higher than 60% ethanol. These results suggest that MAE could be considered a valuable option for CARA extraction since it can be completed quickly using relatively low ethanol concentration and temperature levels.

The CARO levels were dependent on extraction time and ethanol concentration when MAE was applied (Figure 3h). MAE could achieve CARO levels higher than 120 mg/g when the ethanol concentration was raised above 40% and extraction time exceeded 10 min. In the case of UAE and ASE, all three studied parameters affect CARO levels (Figure 2f–h and Figure 4d–f, respectively). It is also observed that the highest possible CARO values do not exceed 100 mg/g extract and are achieved at the intermediate time (6 min) and temperature (45 °C) values and at the highest ethanol concentration. It is obvious that ASE can achieve higher CARO levels than UAE but lower than MAE. In the case of ASE (Figure 4), a narrow area with an ethanol concentration of 50 to 70%, temperature 60 °C, and time more than 5 min yielded CARO levels higher than 120 mg/g, a value similar to that obtained from MAE.

The aforementioned findings show that all extraction processes applied to SWR were mostly affected by ethanol concentration. This fact is in agreement with literature data, which point to the solvent as being crucial for extraction [35,36]. In general, the results of this study reveal that the highest levels of single phenolics recovered from SWR were achieved when high levels of ethanol in the aqueous media were employed. This behavior is similar to that reported in the literature [34]. In their study, Zu et al. [37] reported that the UAE extraction efficiency of RMA from rosemary with water is similar to that extracted with 80% ethanol, while CARA is not extracted in pure water, but it can reach about 100% extraction efficiency at 80% ethanol. On the other hand, higher yield values are achieved when lower ethanol concentrations are applied. UAE is less efficient in extracting CARO and CARA from SWR than MAE and ASE. Generally, a higher ethanol concentration is needed to extract CARA than CARO, regardless of the extraction process applied. On the other hand, UAE should be applied for a longer time to extract CARA than CARO, while the opposite is valid for ASE. Considering these findings, optimization of process parameters can help to reach maximization for all the targeted responses.

The expected optimum values (to achieve the highest desirability among the different possibilities) (Supplementary Figure S1) for each parameter and each extraction process were calculated and reported in Table 2. It was observed that the optimal conditions have a desirability higher than 72%. The predicted optimal conditions for each response were experimentally verified to validate each one generated by the software model. The RSD (relative standard deviation) values vary from 0.99 to 8.92%. As observed, the proximity of predicted and experimental values for each response confirms the validation of the BBD design.

### 2.4. Efficiency of Green Processes on Extraction Yield and Main Phenolic Compounds Contents Compared to Conventional (Soxhlet) Extraction

This study evaluated the extraction efficiency of phenolic compounds from SWR using ASE, MAE, UAE, and Soxhlet extraction techniques. The EY of extracts ranged from 23.2 to 33.6%, showing the highest value in ASE extract and the lowest in Soxhlet extract, although the latter was not significantly different from the other “green” methods (ASE and MAE) (Table 2). Contradictory results are reported in the literature. Palmieri et al. [38] reported that the type of plant matrix plays an important role in extraction efficiency and concluded that Soxhlet gives the highest EY for thyme and hemp, whereas UAE presents the lowest. However, in the case of coriander seed extracts, UAE was the best extraction method. In another study, the Soxhlet method (for 12 h) produced higher EY from dried leaves of *Nepeta spicata* as compared to the ultrasonication technique [39]. Moreover, Rodríguez-Solana et al. [40] reported that the highest EY was achieved by the Soxhlet and ASE techniques.

It seems that the RMA yield does not differ substantially among the three green methods when they run under optimal conditions. Soxhlet should not be recommended for the extraction of RMA since it gives the lowest levels among the methods applied. MAE can be considered the most suitable for RMA since it yielded about 15% more RMA than Soxhlet. On the other hand, all the applied methods, if run under optimal conditions, produce a similar CARA yield. Soxhlet gives a comparable CARO yield with ASE and MAE, while UAE produces the lowest yield among all the tested methods. The results reveal that among the three “green methods”, ASE and MAE can be considered as possible substitutes for Soxhlet in extracting RMA, CARO, and CARA from SWR. Rodríguez-Solana et al. [40] reported that Soxhlet is more efficient than ASE in extracting RMA and CARA from *Mentha piperita*, while the opposite was noted for *Rosmarinus officinalis* L. These observations indicate that plant matrices can play an important role in the efficiency and selectivity of a particular extraction process of phenolics from plant tissues.

### 2.5. Efficiency of Green Processes on TPC, TFCt, and Antioxidant Activity Compared to Soxhlet

**Figure 5:**
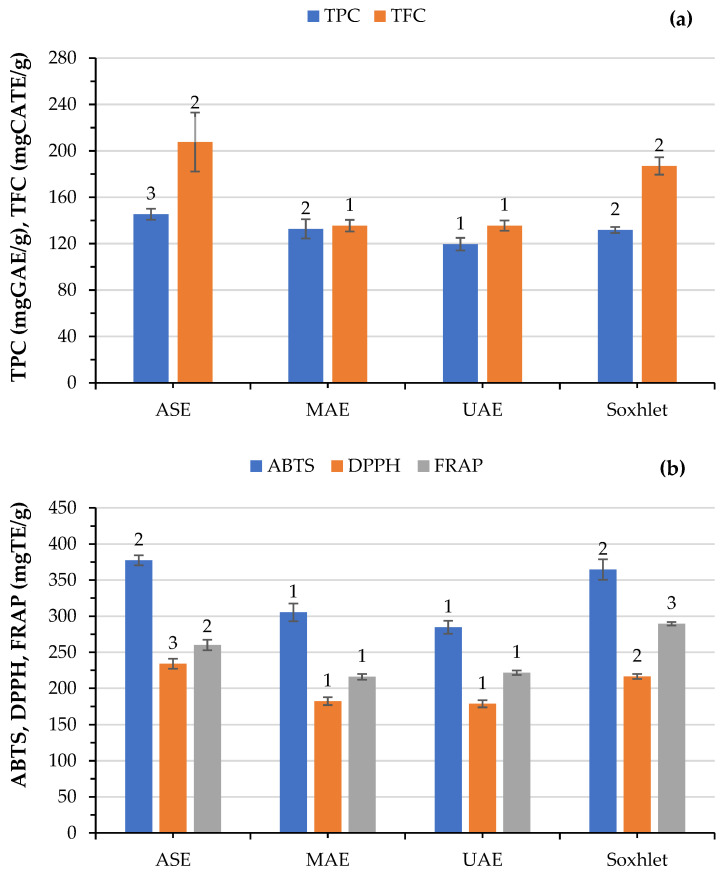
Effect of different extraction processes on (**a**) total phenolic content (TPC), total flavonoid content (TFC), and (**b**) antioxidant activity of the obtained extracts as evaluated by 2,2′-azinobis-(3-ethylbenzothiazoline-6-sulfonic acid (ABTS), 2,2-diphenyl-1-picrylhydrazyl (DPPH), and ferric reducing antioxidant power (FRAP). Bars of the same color capped with the same numbers are not significantly different (*p* > 0.05) from each other, as determined by Duncan’s multiple range test.

The TPC and TFC of the SWR extracts obtained by different extraction processes were evaluated spectrophotometrically using the Folin–Ciocalteu and aluminum chloride methods, respectively. The TPC and TFC results ranged between 119.6 and 145.3 mg GAE/g extract and 135.5 and 207.7 mg CAE/g of extract, respectively (Figure 5a), following the order of ASE > MAE > Soxhlet > UAE. These results showed that ASE provided the highest TPC, whereas UAE had the lowest. Soxhlet and MAE produce extracts with similar TPC values. Similarly, Rodríguez-Solana et al. [40] found that the ASE technique showed the highest values of TPC in the four plants analyzed, including *Rosmarinus officinalis* L. followed by Soxhlet and supercritical fluid (SFE) extraction methods; however, their values were lower than our findings. In the study of Hossain et al. [27], similar TPC values were obtained in *Rosmarinus officinalis* L extract under ASE.

Although ASE produced the extract with the highest TFC value, it does not differ significantly from that obtained by the Soxhlet method; extracts acquired by MAE and UAE showed similar TFC values. In their review, Zhang et al. [36] reported that Soxhlet extraction increases the possibility of thermal degradation of phenolic compounds due to high temperature and long extraction times applied, although this is not always the case. UAE showed the lowest TPC values among all samples. Moreover, it exhibited TPC values similar to MAE and lower than ASE, which operated at much higher temperatures for extraction. Thus, besides temperature and time, other factors can affect the final yield of the phenolic compounds extracted.

The antioxidant activity of the extracts was evaluated by three different assays, namely ABTS, DPPH, and FRAP, to obtain an overall view of the extract’s ability to scavenge electrons or free radicals. These methods are based on different principles of action and are commonly used to study the antioxidant potential of complex samples [41]. It is evident that under optimized conditions, there are significant differences among the extracts from the various extraction processes (Figure 5b). MAE and UAE behave similarly, yielding extracts with similar antioxidant capacity, regardless of the type of assay used, but less than ASE and Soxhlet. ASE produced extracts with higher DPPH but lower FRAP values than Soxhlet, while ABTS values (extracts) were similar between the two extraction methods. In general, compared to the conventional method (Soxhlet), only ASE gives extracts with slightly higher ABTS (3.5%) and DPPH (8.1%) values. Also, Palmieri et al. [38] reported that the Soxhlet extract of thyme was the best in terms of antioxidant activity compared to maceration, UAE, and a rapid solid–liquid dynamic extraction. In addition, they reported that the antioxidant activity of the extract depends on the process as well as on the plant type and the assay applied. Previous experimental findings indicate that the Soxhlet extraction process with ethanol as an exhaustive extraction method was more effective for the recovery of phenolics from sesame than SFE (supercritical fluid extraction) [42]. On the contrary, Karami et al. [43] noted that MAE extracts from licorice root exhibited a higher DPPH radical scavenging capacity than their Soxhlet extraction counterparts.

### 2.6. Energy Consumption and Environmental Impact of Different Extraction Processes

Table 3 lists the input powers, energy consumption, and CO_2_ emission values of various extraction processes. As an environmental protection index, CO_2_ emissions were calculated according to the literature reported by Chen et al. [44]. To acquire 1 kWh from fossil fuel combustion, a total of 800 g of CO_2_ should be emitted into the atmosphere. Among the three extraction methods tested in the present study, UAE exhibited the lowest energy consumption, followed by ASE, MAE, and Soxhlet, due to the shorter extraction time, although it had higher input power compared to ASE and Soxhlet. Although the Soxhlet technique had a relatively low input power for obtaining phenolic extracts, it required a much longer extraction time and the highest energy consumption compared to all other extraction methods tested. Among the ‘green’ extraction methods, MAE had the highest energy consumption due to high input power, while ASE was a more energy-efficient method due to its higher extraction yields. UAE, MAE, and ASE are more environmentally friendly, although their scaling-up processing can be a limiting step in applying these techniques.

Focusing on the environmental implications, the total energy consumption was related to the CO_2_ footprint. In the present study, the CO_2_ emission was dramatically higher for Soxhlet, followed by MAE, ASE, and UAE. In view of its low energy consumption, short extraction time, and low CO_2_ emission, UAE appears to be the most eco-friendly extraction method.

## 3. Materials and Methods

### 3.1. Materials and Chemicals

Aerial parts of fresh rosemary (*Rosmarinus officinalis* L.) were collected in July 2021 from cultivated plants in the experimental field of Hellenic Agricultural Organization—Dimitra, Institute of Plant Breeding and Genetic Resources (Thermi, Thessaloniki, Greece). The plant material, consisting of stalks, leaves, and flowers, was dried at room temperature and stored under controlled environmental conditions (25 °C) until extraction; the maximum storage time before use was 1 month.

Dried plant material (approximately 2 kg) was subjected to steam distillation in a pilot-scale apparatus for approximately 2 h in order to obtain essential oil. The remaining solid residue of rosemary (SWR) after distillation was first sun-dried for 48 h until reaching a moisture content of less than 10% and then ground in a laboratory mill (Retsch, Model ZM 1000, Haan, Germany) to pass through a 0.5 mm sieve. The dried SWR was stored at 4 °C until further analysis.

The analytical reagents 2,2-diphenyl-1-picryhydrazyl (DPPH), 2,2-azinobis-(3-ethylbenzthiazoline-6-sulphonic acid) (ABTS), and 2,4,6-tripyridyl-s-triazine (TPTZ) were purchased from Sigma-Aldrich (Steinheim, Germany). Analytical standards of rosmarinic acid (RMA), gallic acid (GA), and catechin (CAT) were purchased from Extrasynthese (Genay Cedex, France), whereas carnosic acid (CARA) and carnosol (CARO) were obtained from Carbosynth (Berkshire, United Kingdom). Ethanol (96%), which is regarded as a GRAS (generally recognized as safe) solvent for use in the food industry, was employed for the extraction of antioxidants. All the other solvents used for the extraction of phenolic compounds, as well as the chromatographic analysis, were of HPLC or LC-MS grade.

### 3.2. UAE of Phenolic Compounds

UAE of SWR was performed in an ultrasonic homogenizer (BANDELIN, SONOPULS HD 4200, Berlin, Germany) equipped with a GM 4200 generator, an ultrasonic converter UW 100, a SH 100 G booster horn, and a titanium probe TS 103 (diam. 3 mm). Device working conditions employed were as follows: frequency 20 kHz, amplitude 50%, pulse length 2 s, and interval 0.5 s. The powdered SWR material was weighed (0.25 g) into a double-walled extraction tube, and a mixture of ethanol–water (20 mL) with different concentrations was added, according to the experimental design (Table 4). The ultrasound probe was immersed one cm deep into the extraction solvent. During the extraction process, the temperature inside the extractor was constantly monitored with a thermocouple attached to the sonicator system. After the UAE treatment, the extracts were filtered through Whatman No. 1 filter paper lined on a Büchner funnel, and the filtrates were collected in a volumetric flask. Ethanol in the filtrates was evaporated in a rotary evaporator (Heidolph Instruments GmbH & Co. KG, Schwabach, Germany) at 40–60 °C under vacuum. The remaining aqueous extract and the washings were subsequently freeze-dried (Christ, Martin Christ Gefriertrocknungsanlagen GmbH, Germany) for 48 h. The dried extracts were weighed and stored at –25 °C until further analyses. The extraction yield (EY) of SWR was calculated according to the following formula:

(1) $$EY\left(\%\right)=\frac{weight\;of\;freeze-dried\;extract\;(g)}{weight\;of\;pretreated\;SWR\;powder\;(g)}\times 100$$

### 3.3. MAE of Phenolic Compounds

MAE was performed in a commercial microwave-assisted extraction system ETHOS X (Milestone, Sorisole, Italy) equipped with two industrial magnetrons (950 W each) for microwave irradiation and an extraction rotor carrying 15 extraction units. The extraction time and temperature were controlled by the respective software panel. Portions of 0.25 g of powdered SWR materials were mixed with 20 mL of aqueous ethanol solutions in lid-covered TFM vessels (100 mL max. volume) that were placed individually in the respective rotor places. The temperature inside the vessels was monitored via an infrared easyTEMP sensor placed on the bottom of the microwave cavity. Following extraction, using the conditions specified in the experimental design (Table 4), the samples were cooled for 10 min. After completion of the extraction, the extracts were recovered and treated as reported in Section 3.2.

### 3.4. ASE of Phenolic Compounds

Phenolic compounds of SWR were isolated using an ASE device (Dionex Corporation, ASE™ 350 Accelerated Solvent Extractor, Thermo Fisher Scientific Inc., Sunnyvale, CA, USA). The SWR samples (1 g) were mixed with diatomaceous earth (approximately 6 g) as a neutral matrix, and the mixture was placed in a stainless-steel cell (22 mL) equipped with a stainless-steel frit and a cellulose filter (diameter 27 mm, type D28, Thermo Fisher Scientific Inc., Sunnyvale, CA, USA) at the bottom to avoid accumulation of suspended particles in the collection vial. The extraction cells were loaded in the cell tray and were extracted under the conditions described in the experimental design (Table 4), while all other variables were kept constant: 1500 psi, 90 s purge with nitrogen, 5 min as preheating time, 65% volume flush, 3 cycles, and 5 min heating time. Extracts were collected into a 60 mL glass vial with a Teflon septum. The cell was returned to the carousel, and the next sample was extracted. Once completed, the extracts were recovered and treated as reported in Section 3.2. For total ASE extraction time, including 3 applied cycles, preheating and heating time ranged from 18 to 32 min, respectively, whereas the amount of used solvent ranged between 45 and 64 mL per cell, according to the experimental runs in Table 4.

### 3.5. Soxhlet Extraction of Phenolic Compounds

For Soxhlet extractions, about 5 g of dried and powdered SWR was weighed and transferred into a cellulose thimble before being placed into the Soxhlet extractor. The extraction process was performed using 125 mL of 70% ethanol/water (*v*/*v*), applying solid-to-solvent ratio of approximately 1:25. After a period of 4 h, the extract was treated as reported in Section 3.2.

### 3.6. LC-MS Analysis

Major components of the SWR extracts, RMA, CARO, and CARA, were quantified on a Shimadzu Nexera HPLC system (Kyoto, Japan) equipped with a diode array detector and a single quadrupole mass spectrometer (LCMS-2020), which was operated with an electrospray ionization (ESI) interface. Separations were performed on a Poroshell 120 EC-C_18_ analytical column (4.6 × 150 mm, 4 µm), according to a method described in detail by Irakli et al. [45]. Mass acquisitions were performed by targeted selective ion monitoring (SIM) scanning mode. Data acquisition and processing were carried out using the Lab Solutions LC-MS software (Shimadzu, Kyoto, Japan). The main phenolic compounds of the samples were identified by comparing their retention time, UV profile, and mass spectra of unknown peaks with those of authentic standards. For quantitative measurements, a total ion current (TIC) profile was produced in the SIM mode using the calibration curves of corresponding standard solutions, and the results were expressed as mg per g of freeze-dried extract. All the analyses were performed in triplicate.

### 3.7. Determination of Total Phenolic Content, Total Flavonoids Contents and Antioxidant Activity

The determination of total phenolic content (TPC), total flavonoid content (TFC), and antioxidant capacity of the extracts was performed using spectroscopic methods as described by Irakli et al. [45]. The TPC, TFC, and antioxidant activity (ABTS, DPPH, and FRAP) were evaluated as mg gallic acid, catechin, and Trolox equivalents, respectively, per g of dried extract.

### 3.8. Experimental Design and Optimization

The process parameters for each green extraction method (MAE, UAE, and ASE) tested were investigated with response surface methodology (RSM) using a three-level (−1, 0, and +1), three-factor Box–Behnken experimental Design (BBD), consisting of 15 experimental runs carried out, including three replicates on the center point with a random combination of independent variables (Supplementary Tables S1–S3). Each independent variable was chosen based on preliminary experiments. The RSM framework applied to each extraction technique involved the following independent variables: ethanol concentration (X_1_, 0–80%), extraction temperature (X_2_, 30–60 °C), and extraction time (X_3_, 2–10 min) for the UAE technique; ethanol concentration (X_1_, 0–80%), extraction temperature (X_2_, 40–90 °C), and extraction time (X_3_, 3–15 min) for the MAE technique; and ethanol concentration (X_1_, 0–80%), extraction temperature (X_2_, 65–125 °C), and extraction time (X_3_, 3–7 min) for the ASE technique. The selected responses (dependent variables) of each design were EY, RMA, CARO, and CARA. The observed responses at each condition were the mean values of two experiments.

The coded and uncoded values of factors at three levels are explained in Table 4. A full quadratic mathematical model was fitted to each dependent variable obtained from each green extraction technique applied. Model analysis was carried out by employing analysis of variance (ANOVA), and the adequacy and quality of fit of the obtained model were estimated by regression analysis. Only significant terms of the models (*p* ≤ 0.05) were taken into account. Model accuracy was evaluated by lack-of-fit, Fisher test value (F-value), the coefficient of determination (R^2^), the adjusted determination coefficient R^2^_adj_, and the predicted coefficient R^2^_pred_. The design and analysis of the models were performed with the Minitab statistical software (Minitab, version 18, Inc., State College, PA, USA).

Contour plots were generated from the models by varying two variables within the experimental range tested, keeping the third variable constant at the central point. The desirability function of Minitab software was used to optimize extraction conditions (independent factors X_1_, X_2,_ and X_3_), taking into account that for each response (extraction yield, RMA, CARO, and CARA), maximum desirability was needed (i.e., giving a maximum value), and the independent variables were explored within their range of values (between the lowest and the highest level). Optimal conditions obtained from the model were used to fit the experimental data. Under optimized conditions, there were at least two experimental replicates.

### 3.9. Statistical Analysis

Design matrix, fitting, statistical analysis, and optimization of each extraction process (UAE, MAE, and ASE) were calculated by Minitab version 18 (Minitab, Inc., State College, PA, USA). One-way analysis of variance (ANOVA), combined with Duncan’s multiple range test, was used to test for differences among different extraction techniques. The data were tested using SPSS (IBM SPSS Statistics version 26, 2019). For all statistical tests, differences at *p* ≤ 0.05 were considered significant.

## 4. Conclusions

In this study, accelerated solvent extraction (ASE), ultrasonic-assisted extraction (UAE), and microwave-assisted extraction (MAE) were applied to determine the most suitable extraction method for the recovery of phenolic compounds from SWR. The experimental findings pointed to the importance of selecting not only the most suitable extraction method but also the appropriate levels of process parameters that will ensure a high extraction yield of phenolics from SWR combined with a high antioxidant capacity. Among the process variables, the ethanol concentration exerted the greatest influence on the extraction of phenolic compounds from SWR. For each of the tested extraction methods, the content of the main phenolics (RMA, CARO, and CARA) could be maximized by differentiating the ethanol concentration, temperature, and time of extraction. Yet, no single process can be suggested as the best for maximizing the extraction of all three phenolics. Although ASE can be recommended as a novel, eco-friendly, promising, and fully automated extraction technique for high levels of most SWR phenolics, UAE and MAE could also be considered valuable, effective alternatives to Soxhlet extraction. Moreover, UAE and ASE exhibited the highest energy efficiency and the lowest CO_2_ emissions compared with MAE and Soxhlet extraction. Although Soxhlet extraction led to lower power input than UAE and MAE, it required much longer extraction time and higher energy consumption than any other extraction methods used. Considering the antioxidant potential of the phenolic extracts, future studies investigating their stability as well as their bioaccessibility and bioavailability using both in vitro and in vivo approaches are needed.

## Figures and Tables

**Table 1:** Regression equations and analysis of variance (ANOVA) for the modified quadratic models.

Process			Regression Equation in Uncoded Units	R^2^ (%)	R^2^_adj_ (%)	R^2^_pred_ (%)	Lack-of-Fit	
							F	*p*
UAE	EY	=	23.900 − 0.1285 X_1_ + 0.03167 X_2_ + 0.0500 X_3_ + 0.000528 X_1_^2^ + 0.00547 X_1_X_3_	98.1	97.0	93.5	1.72	0.417
	RMA	=	26.23 + 0.3786 X_1_ + 4.98 X_3_ − 0.262 X_3_^2^ − 0.0262 X_1_X_3_	88.0	83.1	70.4	1.51	0.458
	CARO	=	− 60.2 + 0.6521 X_1_ + 3.782 X_2_ * + 6.36 X_3_ * − 0.0418 X_2_^2^ − 0.467 X_3_^2^	96.8	95.1	91.2	1.09	0.558
	CARA	=	121.7 + 1.251 X_1_ + 4.20 X_3_	88.1	86.1	79.9	9.54	0.099
MAE	EY	=	23.541 − 0.09500 X_1_ + 0.0355 X_2_ + 0.2146 X_3_	94.6	93.2	89.4	3.98	0.217
	RMA	=	27.96 + 0.1594 X_1_ + 0.701 X_2_ * − 0.00554 X_2_^2^	94.8	93.4	90.0	1.25	0.522
	CARO	=	22.39 + 2.198 X_1_ + 1.238 X_3_ − 0.01526 X_1_^2^	95.4	94.2	90.9	17.14	0.056
	CARA	=	40.7 + 5.267 X_1_ + 0.959 X_2_ * − 11.03 X_3_ * + 0.939 X_3_^2^ − 0.02559 X_1_X_2_ − 0.0876 X_1_X_3_	97.8	96.1	87.6	10.93	0.086
ASE	EY	=	31.91 − 0.0996 X_1_ + 0.837 X_3_	89.1	87.3	80.8	9.54	0.099
	RMA	=	45.95 + 0.11058 X_1_ + 0.0418 X_2_	94.3	93.4	90.3	4.18	0.208
	CARO	=	86.6 + 1.885 X_1_ − 1.062 X_2_ * + 6.84 X_3_ * − 0.020859 X_1_^2^ + 0.00716 X_2_^2^ − 1.391 X_3_^2^ − 0.00728 X_1_X_2_ + 0.1926 X_1_X_3_	99.4	98.6	94.5	9.36	0.099
	CARA	=	221.1 + 2.567 X_1_ − 2.66 X_2_ − 0.02196 X_12_ + 0.01642 X_2_^2^ + 0.01295 X_1_X_2_	98.8	98.1	97.3	1.08	0.561

X_1_, ethanol concentration; X_2_, extraction temperature; X_3_, extraction time; MAE, microwave-assisted extraction; UAE, ultrasound-assisted extraction; ASE, accelerated solvent extraction; EY, extraction yield; RMA, rosmarinic acid; CARO, carnosol; and CARA, carnosic acid. * Only significant equation terms were reported except for main terms if coefficients that explain quadratic or interaction effects are significant (*p* ≤ 0.05). Equations are obtained in terms of uncoded variables (real values).

**Table 2:** Optimal extraction conditions, composite desirability, predicted and experimentally determined values based on the proposed models for describing the extraction outcome of phenolics from SWR with three different processes.

	Process	X_1_	X_2_	X_3_	Composite Desirability	EY (%)	RMA (mg/g)	CARO (mg/g)	CARA (mg/g)
Predicted	ASE	59.0	125.0	7	0.72	31.90 ± 0.58 (3.56)	57.78 ± 0.43 (4.66)	109.83 ± 2.31 (6.51)	315.96 ± 6.81 (8.92)
	MAE	67.4	69.8	15	0.75	22.83 ± 0.42 (3.65)	60.63 ± 0.60 (2.01)	119.77 ± 4.04 (8.19)	299.40 ± 11.40 (1.79)
	UAE	80.0	50.6	10	0.79	23.48 ± 0.27 (5.74)	59.19 ± 2.69 (6.60)	93.18 ± 3.06 (0.99)	263.8 ± 8.93 (8.75)
Experimental	ASE					33.55 ± 2.29 ^2^	54.10 ± 3.67 ^1,2^	100.16 ± 1.54 ^1,2^	278.46 ± 4.24 ^1^
	MAE					24.04 ± 1.57 ^1^	58.93 ± 1.65 ^2^	106.65 ± 3.50 ^2^	307.08 ± 30.21 ^1^
	UAE					25.47 ± 1.27 ^1^	53.91 ± 3.85 ^1,2^	94.50 ± 5.57 ^1^	298.59 ± 25.52 ^1^
	Soxhlet					23.18 ± 0.23 ^1^	51.40 ± 0.50 ^1^	102.39 ± 1.21 ^2^	306.17 ± 16.49 ^1^

MAE, UAE, ASE, microwave-assisted extraction, ultrasound-assisted extraction, and accelerated solvent extraction, respectively. EY, extraction yield; RMA, rosmarinic acid; CARO, carnosol; CARA, carnosic acid; and RSD, relative standard deviation (in parentheses). Different superscript numbers in the same column indicate differences (*p* ≤ 0.05) amongst the means, as determined by Duncan’s multiple range test. The experimental data are means of three independent replicates  ±  standard deviation.

**Table 3:** Energy consumption and environmental impact of different extraction processes.

Extraction Method	Input Power (KW/g Extract)	Extraction Time (min)	Energy Consumption (KWh/g Extract)	CO_2_ Emissions (kg/g Extract)
UAE	3.14	10	0.52	0.42
MAE	9.98	15	2.45	2.00
ASE	1.49	32	0.80	0.64
Soxhlet	1.94	240	7.78	6.21

**Table 4:** Coded and actual levels of independent variables used for ultrasound-assisted extraction (UAE), microwave-assisted extraction (MAE), and accelerated solvent extraction (ASE).

	Coded Values		
	−1	0	+1
UAE			
Ethanol concentration (%)	0	40	80
Extraction temperature (°C)	30	45	60
Extraction time (min)	2	6	10
MAE			
Ethanol concentration (%)	0	40	80
Extraction temperature (°C)	40	65	90
Extraction time (min)	3	9	15
ASE			
Ethanol concentration (%)	0	40	80
Extraction temperature (°C)	65	95	125
Extraction time (min)	3	5	7

Solid-to-solvent ratio was constant: 1:80 (UAE and MAE) or 1:60 (ASE).

## Data Availability

Data are contained within the article and Supplementary Figure.

## Supplementary Materials

The following supporting information can be downloaded at https://www.mdpi.com/article/10.3390/molecules28186669/s1. Figure S1: Overlaid contour plots; Table S1: UAE experimental design; Table S2: MAE experimental design; and Table S3: ASE experimental design.

## Sample Availability

Samples of the compounds are available from the authors.
