# Supplementary material for: Sustainable Recovery of Phenolic Compounds from Distilled Rosemary By-Product Using Green Extraction Methods: Optimization, Comparison, and Antioxidant Activity
Source: Molecules. 2023 Sep 17;28(18):6669. doi: 10.3390/molecules28186669 (PMC10537096; doi:10.3390/molecules28186669)
Supplement: Supplementary file 1 [file molecules-28-06669-s001.zip › molecules-2611085-supplementary.pdf]

Table S1. Experimental values for extraction yield (EY) and three main target compounds: rosmarinic acid (RMA), carnosol (CARO) and carnosic acid (CARA) extracted from SWR by ultrasound-assisted extraction in the function of coded values of the extraction factors.

Run	Independent variables			Target responses			
	Ethanol conc. (%)	Temp. extraction (◦C)	Time extraction (min)	EY (%)	RMA (mg/g)	CARO (mg/g)	CARA (mg/g)
1	0 (−1)	30 (−1)[a]	6 (0)	19.5	47.8	20.9	81.6
2	80 (1)	30 (−1)	6 (0)	15.0	60.2	70.0	185.0
3	0 (−1)	60 (1)	6 (0)	20.0	49.0	20.3	48.0
4	80 (1)	60 (1)	6 (0)	16.2	70.2	75.0	156.5
5	0 (−1)	45 (0)	2 (−1)	19.6	32.0	23.6	51.0
6	80 (1)	45 (0)	2 (−1)	13.4	59.0	67.9	145
7	0 (−1)	45 (0)	10 (1)	19.6	49.9	20.9	60.5
8	80 (1)	45 (0)	10 (1)	16..9	60.2	81.6	155.0
9	40 (0)	30 (−1)	2 (−1)	14.7	51.1	37.6	85.0
10	40 (0)	60 (1)	2 (−1)	15.7	50.6	41.2	75.2
11	40 (0)	30 (−1)	10 (1)	17.2	53.1	48.7	140.0
12	40 (0)	60 (1)	10 (1)	18.3	54.9	43.1	135.0
13	40 (0)	45 (0)	6 (0)	17.1	53.3	64.2	120
14	40 (0)	45 (0)	6 (0)	17.0	57.0	60.8	115.3
15	40 (0)	45 (0)	6 (0)	16.6	51.4	55.3	126.4

[a] coded level of independent variables

Table S2. Experimental values for extraction yield (EY) and three main target compounds: rosmarinic acid (RMA), carnosol (CARO) and carnosic acid (CARA) extracted from SWR by microwave-assisted extraction in the function of coded values of the extraction factors.

Run	Independent variables			Target responses			
	Ethanol conc. (%)	Temp. extraction (◦C)	Time extraction (min)	EY (%)	RMA (mg/g)	CARO (mg/g)	CARA (mg/g)
1	0 (−1)	40 (−1)[a]	9 (0)	23.5	46.4	21.0	65.4
2	80 (1)	40 (−1)	9 (0)	14.4	60.5	102.7	316.6
3	0 (−1)	90 (1)	9 (0)	24.4	44.2	38.6	109.2
4	80 (1)	90 (1)	9 (0)	17.4	59.4	122.0	258.1
5	0 (−1)	65 (0)	3 (−1)	22.3	49.0	21.3	75.0
6	80 (1)	65 (0)	3 (−1)	14.2	61.2	103.1	367.4
7	0 (−1)	65 (0)	15 (1)	25.0	52.0	53.2	141.3
8	80 (1)	65 (0)	15 (1)	18.8	61.4	119.1	349.6
9	40 (0)	40 (−1)	3 (−1)	18.3	53.3	94.8	191.1
10	40 (0)	90 (1)	3 (−1)	20.9	52.8	95.3	214.6
11	40 (0)	40 (−1)	15 (1)	20.8	53.7	106.7	246.1
12	40 (0)	90 (1)	15 (1)	21.4	53.7	94.9	224.5
13	40 (0)	65 (0)	9 (0)	19.0	57.1	95.5	199.2
14	40 (0)	65 (0)	9 (0)	19.4	58.5	98.1	205.3
15	40 (0)	65 (0)	9 (0)	19.9	56.0	93.8	192.9

[a] coded level of independent variables

Table S3. Experimental values for extraction yield (EY) and three main target compounds: rosmarinic acid (RMA), carnosol (CARO) and carnosic acid (CARA) extracted from SWR by accelerated-solvent extraction in the function of coded values of the extraction factors.

Run	Independent variables			Target responses			
	Ethanol conc. (%)	Temp. extraction (◦C)	Time extraction (min)	EY (%)	RMA (mg/g)	CARO (mg/g)	CARA (mg/g)
1	0 (−1)	65 (−1)[a]	5 (0)	31.4	47.5	46.4	117.0
2	80 (1)	65 (−1)	5 (0)	23.0	56.3	100.9	250.9
3	0 (−1)	125 (1)	5 (0)	33.1	51.3	68.1	147.3
4	80 (1)	125 (1)	5 (0)	25.3	60.9	87.6	343.3
5	0 (−1)	95 (0)	3 (−1)	28.8	49.2	57.9	126.0
6	80 (1)	95 (0)	3 (−1)	22.5	58.1	68.1	272.5
7	0 (−1)	95 (0)	7 (1)	35.8	50.1	28.6	105.0
8	80 (1)	95 (0)	7 (1)	26.5	58.2	100.4	281.5
9	40 (0)	65 (−1)	3 (−1)	26.9	54.4	105.1	229.9
10	40 (0)	125 (1)	3 (−1)	27.4	54.7	98.4	278.2
11	40 (0)	65 (−1)	7 (1)	27.6	54.3	105.4	206.8
12	40 (0)	125 (1)	7 (1)	29.0	55.7	105.4	269.8
13	40 (0)	95 (0)	5 (0)	27.7	55.3	102.8	213.7
14	40 (0)	95 (0)	5 (0)	28.4	54.7	101.5	247.2
15	40 (0)	95 (0)	5 (0)	28.5	54.4	103.8	226.9

[a] coded level of independent variables

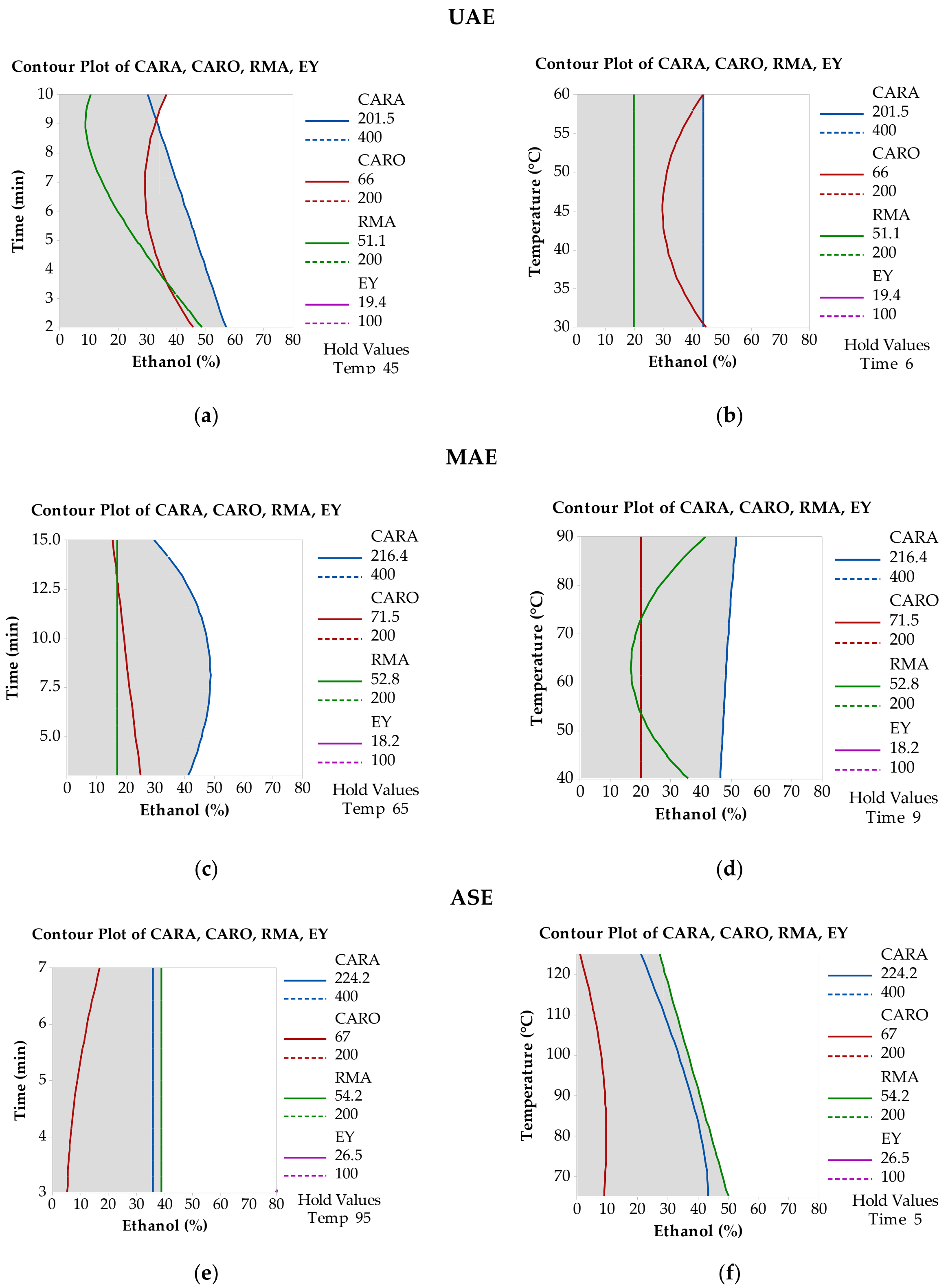

Supplementary Figure S1. Overlaid contour plots showing the effect of the interaction of ethanol concentration with time/temperature of extraction on four responses. Contour plots are drawn: in the case of yield having as lower bound its minimum value and as upper boundary 100%; for the rest of variables having as lower bound the minimum value plus half of the difference between the maximum and the minimum value and as upper bound values of 200 for RMA and CARO. and 400 for CARA regardless of the extraction method (UAE. MAE. ASE). White areas are showing feasible regions where all responses (EY. RMA. CARO and CARA) are within their boundaries.
